# Evolutionary Pets: Offspring Numbers Reveal Speciation Process in Domesticated Chickens

**DOI:** 10.1371/journal.pone.0041453

**Published:** 2012-08-06

**Authors:** Inga Tiemann, Gerd Rehkämper

**Affiliations:** 1 Bruno-Dürigen Institute, Poultry Research Centre, Rommerskirchen, Germany; 2 Research Group Comparative Neurobiology and Evolutionary Research, Institute of Anatomy, Heinrich-Heine-University, Düsseldorf, Germany; University of Georgia, United States of America

## Abstract

Since Darwin, the nature of the relationship between evolution and domestication has been debated. Evolution offers different mechanisms of selection that lead to adaptation and may end in the origin of new species as defined by the biological species concept. Domestication has given rise to numerous breeds in almost every domesticated species, including chickens. At the same time, so-called artificial selection seems to exclude mechanisms of sexual selection by the animals themselves. We want to forward the question to the animal itself: With whom do you reproduce successfully? This study focused on the sexual behavior of the domestic chicken *Gallus gallus* f.dom., particularly the White Crested Polish breed. Experiments on mate choice and the observation of fertilization and hatching rates of mixed-breeding groups revealed breed-specific preferences. In breeding groups containing White Crested Polish and a comparative breed, more purebred chicks hatched than hybrids (number of eggs collected: 1059). Mating was possible in equal shares, but in relation to the number of eggs collected, purebred offspring (62.75%±7.10%, M±SE) hatched to a greater extend compared to hybrid offspring (28.75%±15.32%, M±SE). These data demonstrate that the mechanism of sexual selection is still present in domestic chicken breeds, which includes the alteration of gene frequencies typical for domestication and evolutionary speciation. Due to selection and mate choice we state that breeding in principle can generate new species. Therefore, we see domestication as an evolutionary process that integrates human interests of animal breeding with innate mate choice by the animal.

## Introduction

In the book “Speciation” [1], the authors Coyne and Orr give a short historical overview about speciation research demonstrating that adaptation, sexual selection and reproductive isolation are major catchwords closely related to species concepts. In the past, different species concepts were established and in the course of time further discussed and modified. The main ideologies of those concepts are indicated in their titles such as genotypic species concept, recognition species concept, cohesion species concept, evolutionary species concept, ecological species concept, and phylogenetic species concept.

These concepts do not abrogate the value of the biological species concept (BSC) as proposed by initially Ray (1686–1704) and elaborated by Mayr [2]. According to this concept, species are groups of individuals that can interbreed and which are reproductively isolated from other such populations. In general, the BSC meets all requirements of modern species concepts [3] and is, therefore, still a useful heuristic tool for evolutionary research [1], [4]. Its value lies in the ability to use the concept to formulate hypotheses that can be tested in experiments using living animals. For example, in the context of sexual selection, females were hypothesized and later shown to be the “choosy” sex [5].

Strongly connected with the choosy sex are observations on non-random, so-called assortative mating. Assortative mating reduces the intraspecific gene flow which results in phenotypic divergence and may end up in a reproductive isolated population [6]. Several examples are described in Bateson's book on mate choice [7] such as the Snow Goose *Anser caerulescens caerulescens* [8] and the mallard *Anas platyrhynchos* [9]. For Snow Geese it has been shown that mating is assortative concerning the two colour morphs, white and blue, based on sexual imprinting of the goslings. Kasper Hauser experiments on male and female Mallards revealed innate factors influencing mate choice [10]. Both observations are still discussed according to their evolutionary value, the prevention of interspecific hybridization, and their driving forces: species recognition based on early learning or genetic predispositions [11].

Studies applying the BSC commonly used *Drosophila* [12] and different fish species [13] for evolutionary experiments. More seldom homoiothermic vertebrates are studied, such as zebra finches [14] and laboratory rodents e.g. mice [15]. We have a special interest in domesticated animals and believe them to be a useful tool for evolutionary research and investigating speciation. The use of domesticated animals in the study of evolution has a long tradition and can be traced back to Darwin [16], [17] and his observation of domestic poultry. Among the many authors who studied domestic animals under evolutionary aspects, Wright's shifting balance theory of evolution has to be mentioned. It was based on experiments with hooded rats and guinea pigs, and on the analysis of the Shorthorn cattle pedigrees; all of them domesticated animals [18], [19]. Wright had no concerns in paralleling domestication and evolution whereas others are more reserved.

In Germany, Herre and Röhrs [20] founded a school of domestication research in 1947 and propagated domestication as being a suitable model of evolution. But at the same time, the authors did not see a *complete* congruence between domestication and evolution. They stressed that domestication does not lead to new species since sexual attractiveness between domestic animals and their wild living relatives would still exist. As a consequence, domestic animals would not represent a stable population since crossings would disperse their domestic population upon mating with their wild relatives. However, the authors did not test their hypothesis experimentally.

Recently, Edward O. Price [21] compared domestication and evolution, and formulated supporting arguments such as the differentiation of the wild and domestic phenotype and their adaptation to man on a genetic base. However, Trevor Price [11] sees two restrictions that prevent domestic animals from being an ideal model of evolution. First, he argues that animal breeders are specifically looking for new traits which contrast natural selection. Second, human breeders are said to select for traits which would be deleterious for a wild living animal.

In our study we observed domestic poultry, using the domesticated chicken as an animal model. The reason for this is that chickens as well as pigeons have undergone a remarkable diversification. It is assumed that *Gallus gallus gallus* Linné 1758, and/or *G. g. spadiceus* (Bonnaterre, 1791) directly originate from the population which also gave rise to the domesticated chicken about 8.000 years ago [22], or even long before the archaeological dating of domestication [23]. Despite the discussion of their origin, it is undisputed that domestic chickens are found all over the world in more than 500 economical and fancy breeds [24], [25]. Herre and Röhrs [20] have proposed to subsume all domesticated chicken breeds under the scientific name *Gallus gallus* forma domestica (f.d.), but most authors use *G. g. domesticus*.

The species status of *Gallus sonneratii* and *Gallus gallus* is defined by a list of isolation mechanisms: geographic isolation, behavioural isolation, and genetic isolation [26]. According to current systematic terminology, neither the diverse subpopulations of the wild *Gallus gallus* nor the domestic population or parts of it (breeds) are seen to have reached the status of a species of its own [27]. We follow the argumentation of Bleed [28] who coined the expression of a ‘human niche’ to which domesticated plants as well as animals have adapted. This niche concept requires adaptations which are physical, behavioural and include human action such as habitat manipulations and selection patterns. Within this niche or habitat, domestic breeds are kept in allopatry to avoid interbreed hybridization. In general, these criteria are associated with speciation, first and foremost geographic isolation [2] but also adaptation to different habitats [29].

According to the theoretical background, our research addresses two questions: 1. Has a distinctive chicken breed reached species status in terms of the BSC? and, 2. Can the analysis of social and/or reproductive behaviour of the domestic chicken help to understand speciation and evolution?

**Figure 1:**
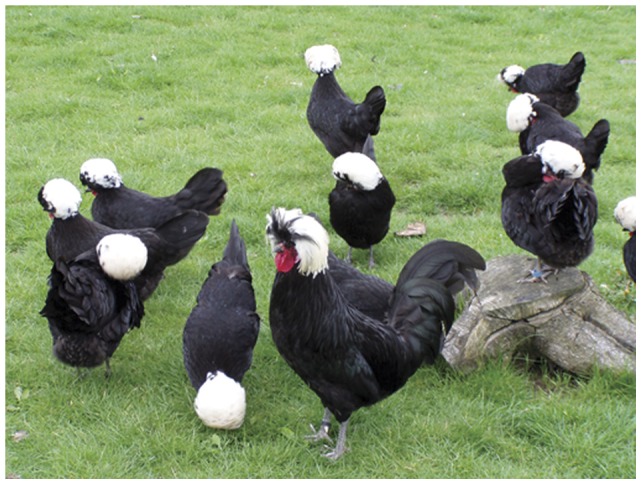
A breeding group of White Crested Polish chickens.

**Figure 2:**
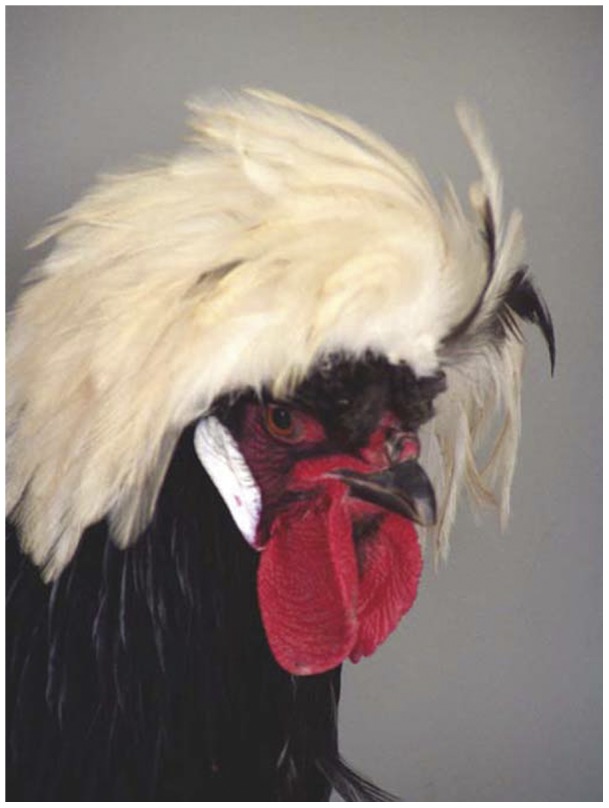
Portrait of a cock of the breed White Crested Polish.

To answer these questions we designed an experiment, using a special breed of the domestic chicken, which we have investigated in previous studies, the Polish (US) or Poland (GB) chicken (more detailed, the White Crested Black Polish, WCP, figures 1 and 2). Darwin, as early as 1868, published a drawing highlighting the outer appearance of the Polish breed. Today the breed can be traced back to roman times [30]. In this breed the comb has been drastically reduced and is hardly visible in the male as well as in the female. Striking is the crest, which is a feathery balloon situated upon a bony protuberance of the skull. Also striking is the extraordinary large brain and characteristic brain composition [31], [32]. Particularly, the crest is the breeder's focus, who selects for this trait according to a standard of perfection, for example, the “Standard of Perfection of the American Poultry Association (US)”, the “British Poultry Standards (GB)”, and “Rassegeflügelstandard für Europa (Germany)”.

Bringing together domestication and evolution, we have to define the role of sexual selection in order to formulate our hypothesis. One possible definition of evolution is given by the alteration in gene (allele) frequencies [33]. In sexually reproducing organisms such as chickens, mate choice plays an important role [34]–[39] and female choice might be more important than male choice in causing sexual selection [5], [40]–[42].

Studies on sexual selection need to be run with an exceedingly careful experimental design. In a different study with chickens, Lill [43], [44] reported on breed-specific preferences of hens for cocks of their own breed in the context of preferential mating. Unfortunately, the experimental design of the study did not allow to exclude learning effects since animals were raised separately and, therefore, showed different life-histories [45], [46]. Filial imprinting [47] including sexual imprinting [48], [49] might be among the strongest driving forces in mate choice. To prevent unbalanced influences on the sexual behaviour in our study, all test chickens were raised in one group sharing the same environmental experiences including social contacts. For this reason, we (a) hatched the same number of chicks per breed, (b) kept equal numbers of males and females per breed, and (c) raised all chicks of both breeds in one single group. All the mechanisms should ensure that any experiences either with the opposite sex or the comparative breed are balanced. This experimental design is a major improvement compared to earlier studies on assortative mating [46], [50].

Thus, we investigated if female WCP prefer WCP cocks as mates in comparison with cocks of two other breeds (figure 1 and 2, table 1). Particularly, we looked for successful mating which resulted in countable offspring numbers as a quantitative value. Is the relative advantage of purebred mating in terms of numbers of offspring higher than those offspring numbers indicating hybridization? If the answer is “no” then this would support the argument that domestication cannot be seen as an ongoing evolutionary process. However, if the answer is “yes” this would indicate that WCP are a freely inbreeding population based on sexual selection and reproductive isolation, which means that they could be regarded as a species as defined by the BSC.

**Table 1:** Pairing scheme of the mating experiments (x, 0 =  male, 0, x =  female, WCP White Crested Polish, RL Red Leghorn, LSL Lohmann Selected Leghorn).

Group 1a	0,3 WCP 0,3 RL	1,0 WCP
Group 1b	0,3 WCP 0,3 RL	1,0 WCP
Group 2a	0,3 WCP 0,3 RL	1,0 RL
Group 2b	0,3 WCP 0,3 RL	1,0 RL
Group 3a	0,3 WCP 0,3 LSL	1,0 WCP
Group 3b	0,3 WCP 0,3 LSL	1,0 WCP
Group 4a	0,3 WCP 0,3 LSL	1,0 LSL
Group 4b	0,3 WCP 0,3 LSL	1,0 LSL

Our results accomplished the proposed intention and reveal that speciation processes can be found among domestication animals.

## Results

**Figure 3:**
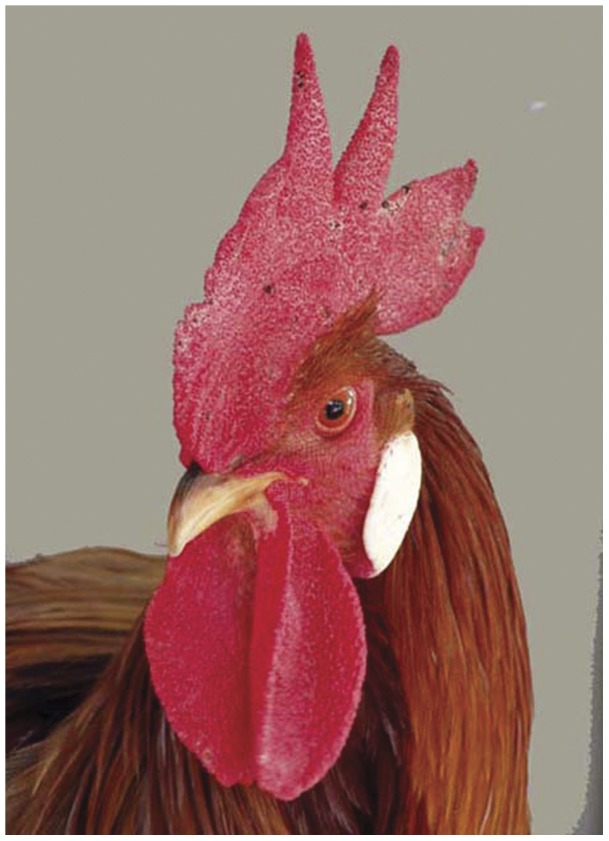
Fertilization rates of the breeding groups (stars indicate significant differences between performances after hybrid and purebred mating, respectively).

**Figure 4:**
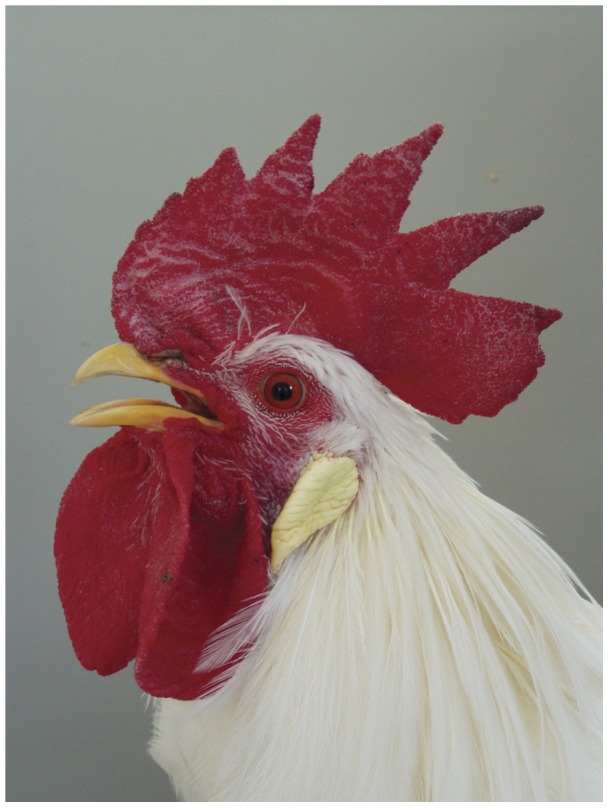
Hatching rates of the breeding groups (stars indicate significant differences between performances after hybrid and purebred mating, respectively).

Fertilization and hatching rates are given in table 2 and visualized in figure 3 and figure 4, respectively. In the first year with WCP and RL, WCP hens laid a total of 247 eggs. Pure-breed mating resulted in higher fertilization and hatching rates compared to hybrid mating. This was statistically significant for WCP hens in groups 1a+b which were mated with a WCP cock compared to WCP hens in groups 2a+b which were mated with a RL cock, for the fertilization rates χ^2^ (1, *n* = 247)  = 9.846, two-tailed *p*≤.01, as well as for the corresponding hatching rates χ^2^ (1, *n* = 247)  = 7.563, two-tailed *p*≤.01.

**Table 2:** Fertilization and hatching rates of the breeds WCP, RL and LSL of the different mating groups.

Breed (female)	Mating (male)	Laid eggs (n)	Fertilized eggs (n)	Fertilization rate (transformed)	Hatching rate (transformed)	Chicks (n)
WCP 1	internal (WCP) Groups 1a+b	157	127	.81 (64.16)	.75 (60.00)	117
	external (RL) Groups 2a+b	90	41	.46 (42.71)	.43 (40.98)	39
RL	internal (RL) Groups 2a+b	116	63	.54 (47.29)	.49 (44.43)	57
	external (WCP) Groups 1a+b	81	6	.07 (15.34)	.07 (15.34)	6
WCP 2	internal (WCP) Groups 3a+b	50	36	.72 (58.05)	.52 (46.15)	26
	external (LSL) Groups 4a+b	10	1	.10 (18.43)	0 (9.10)	0
LSL	internal (LSL) Groups 4a+b	323	279	.86 (68.03)	.75 (60.00)	241
	external (WCP) Groups 3a+b	232	176	.76 (60.67)	.65 (53.73)	151

Similar results were obtained in the comparison of fertilization and hatching rates of RL hens in groups 2a+b with a cock of the same breed and of groups 1a+b after mating with a WCP cock. RL hens laid a total of 197 eggs and again, pure-breed fertilization rates were superior over fertilization rates resulting from hybrid mating with χ^2^ (1, *n* = 197)  = 20.252, two-tailed *p*≤.01, as well as for the corresponding hatching rates, χ^2^ (1, *n* = 197)  = 44.43, two-tailed *p*≤.01.

In the second year, WCP and LSL were compared. WCP laid a total of 60 eggs in the observation period. The fertilization rate differs significantly between pure-breed and hybrid mating, χ^2^ (1, *n* = 60)  = 3.771, two-tailed *p*≤.05. The hatching rate did not reach significance, χ^2^ (1, *n* = 60)  = 3.348, two-tailed *p*≤.067, due to former Bartlett's transformation (see methods for statistical details).

LSL hens laid a total of 555 eggs. Fertilization rate, χ^2^ (1, *n* = 555)  = 2.898, two-tailed *p* = .09, and hatching rate, χ^2^ (1, *n* = 555)  = 1.922, two-tailed *p* = .17, both did not show any significant advantages for pure-breed mating compared to hybrid mating.

## Discussion

### The status of WCP in terms of the BSC

The rationale for this study was firstly to see whether a distinct breed of the domestic chicken (WCP) shows assortative mating and a preference for purebred pairings which are seen as primers of further speciation processes. The data support this hypothesis and are in line with two previous studies which demonstrated that WCP chicks show breed-specific flocking [51] and that mature WCP hens spend significantly more time with cocks of their own breed than with cocks of comparative breeds [52]. However, the experimental design of previous studies did not allow for copulation and, therefore, actual reproduction of offspring. This has been in the focus of the present investigation. Individual differences in mating behaviour might appear but on average, mating results in purebred offspring to a larger extent.

Integrating behavioural data from previous studies and reproductive data of the present study, we propose that WCPs should be identified as a freely inbreeding population which is separated from other such populations. This characterization fulfils the criteria of the biological species concept [2]. Wu [53] has discussed the BSC and proposed a four stage model of speciation. We support the idea of characterizing speciation as a gradual process and would mark LSL to stage I and WCP to be close to stage III of Wu's stage model of speciation [53]. However, reproductive isolation is not an all-or-none phenomenon and a gene flow could still take place.

Price [11] put forward two arguments that prevent him from completely paralleling evolution and domestication. The first argument was that the breeder seeks for a specific trait and nature does not. Both, natural selection and breeder's choice are based on new traits that appear by chance. The only difference is that humans as breeders know about the reason to select for a specific trait, whereas in nature it is not always obvious to the observer which trait have been selected for what reason. The second argument of Price [11] was that man selects for traits that would severely impair an animal in the wild. At this point we clearly have to state that domestic animals do not live in the wild, moreover, the ‘natural’ environment of domestic animals is close proximity to humans. They have, in other words, successfully conquered a new ecological niche: man and his farm, as Rubin et al. [54] mention it in the introduction of their paper. Seen from this point of view, both arguments of Price [11] do not restrain a far reaching parallelism between evolution in the wild and evolution within domestication.

### Female choice and the problem of low fertilization rates

We assume that female choice can, first and foremost, explain these data. This is in accordance with the large amount of literature that describe females as the “choosy” sex *inter alia* because of the higher investment of females in producing megagametes (eggs) and parental care [5].

The problem of low fertilization and hatching rates after heterospecific mating in our experiment has to be discussed. Although, we did not record the number of copulations or the amount of sperm transferred, successful reproduction did take place to a considerable degree. To explain those cases in which no successful offspring was produced, there is a wide range of postcopulatory, pre- and postzygotic barriers known to prevent successful development [55], [56]. Unfortunately our ethological study cannot contribute to this field of research which requires microscopic techniques for the analysis of eggs and parental generation.

### Is mate choice inborn or learned?

One crucial argument of the nature/nurture debate is whether evolution is based on heritable traits or influenced by individual life history and learned behaviour, including imprinting.

The relevance of learning and experience and their influence on mate choice has been investigated by several researchers [14], [57]–[59]. One of many examples might be found in the North American Brown-headed Cowbird (*Molothrus ater*) who has been shown to use more than 140 species successfully for its brood parasitism [11]. Although, offspring of this species is not brought up by their genetic parents; sexual mates will still be conspecifics. An explanation for their successful mating system, without previous filial or sexual imprinting, could be self-referent phenotype matching [60]. Other studies with domestic chicks reveal the complexity of filial imprinting in which genetic predispositions prepare following imprinting processes [47]. Among Zebra Finches, in the same sex parts of mate choice might be innate whereas others might be learned [14]. One of the findings these studies have in common is, that learning processes are supposed to have an innate and, therefore, genetic background.

Furthermore, we would like to point out that altricial birds do not share the same brooding environment as precocial birds. The balance of innate and learned factors within mate choice might shift to either side depending on whether the animals in focus belong to one or the other biological category.

Independent of the brood type, any experience with peers will also influence the individual's mate choice. Since Clayton [14] has shown that the presence of brothers can influence sexual imprinting of females, we kept equal numbers of males and females of both breeds, the WCP and the comparative breed, until chickens reached sexual maturity. We also reduced the individual's influence, such as a low/high condition [61], on the data set by independent breeding groups in two consecutive years. Nevertheless, we found breed-specific preferences for the cocks of the own breed and for purebred mating. Because of this, we state that assortative mating and the establishment of the relative reproductive isolation between the chicken breeds tested here are heavily influenced by heritable traits.

## Methods

**Figure 5:**
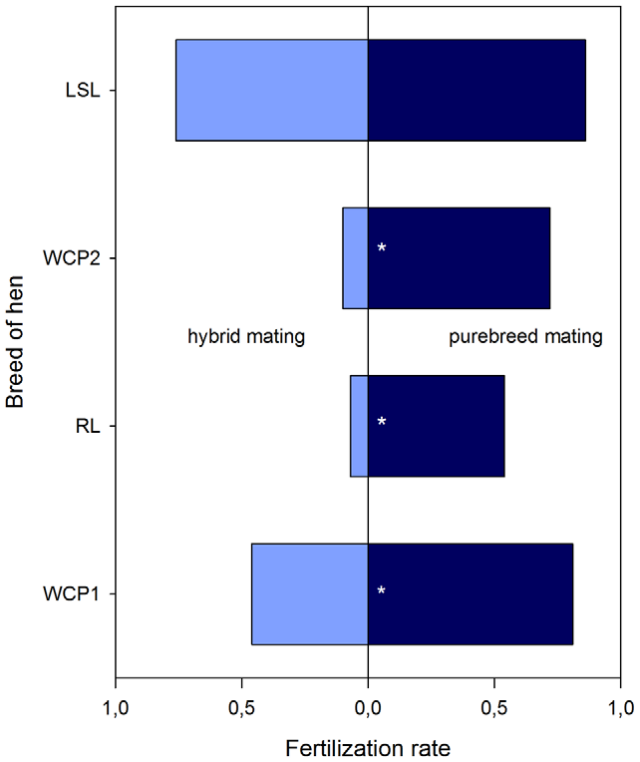
Portrait of a cock of the breed Red Leghorn.

**Figure 6:**
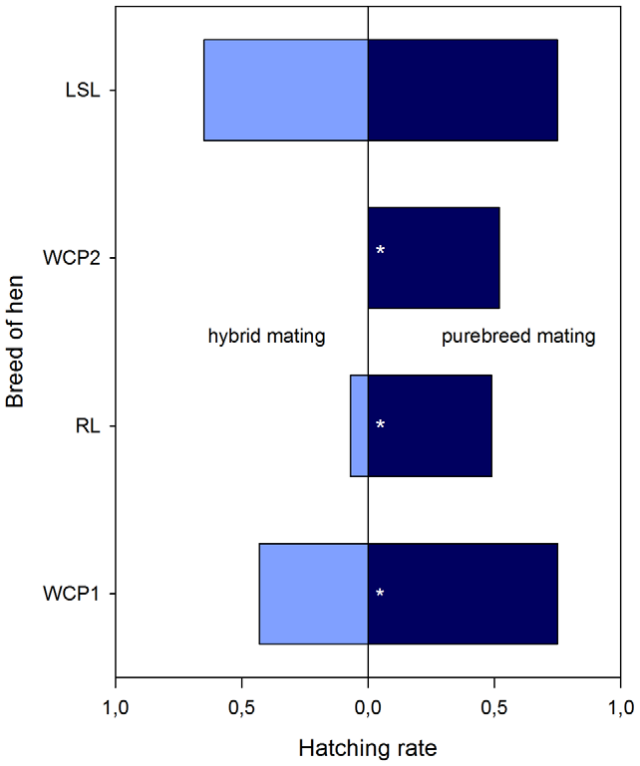
Portrait of a cock of the breed Lohmann Selected Leghorn Classic.

Four mixed breeding groups were established, each of the four breeding groups contained three female WCPs and three Red Leghorns (RL, year 1, figure 5) or Lohmann Selected Leghorns (LSL, year 2; see figure 6). In two of the four groups a WCP cock joined the hens; in the other two groups a cock of the comparative breed was added (group a and b in table 1). All animals were incubated, hatched and raised together to ensure that life-history and social as well as external experiences did not influence the collected samples. Moreover, animal numbers in terms of sex and breed were kept equal throughout the experimental prehistory. Each group was kept in a small chicken house (W×D×H: 120×120×190 cm) containing perches and a brood nest. Each chicken house was located on a meadow of 250 m^2^. Water, commercial chicken food, and grit were provided ad libitum.

The collection of the eggs was started ten days after the establishment of the breeding groups to ensure fertilization. Collected eggs were easily assigned to the breeds investigated. The eggs were stored at room temperature, and every ten days a new set of eggs was incubated (Temp. 37.8C°, humidity 53%, rate of rotation was three times per day; duration of cooling was 25 min per day). After ten days of incubation eggs were candled to record fertilization rate. Unfertilized eggs were rejected and all others were incubated eight more days. At day 18 the eggs were moved into the hatching partition of the incubator (Temp. 37.5C°, humidity 73%, no rotation or cooling). Chicks usually hatched at day 21 but eggs were left in the hatching partition to day 23 because late-hatchers were possible.

The proportions of fertilization rate and hatching rate were calculated using the total number of laid and incubated eggs of each experimental group. We preferred to compare proportions rather than absolute numbers since the egg laying capacity of the commercial breed LSL is high compared to traditional chicken breeds. Influences by individual cocks were reduced by pooling data of the same category of pairing (WCP-WCP, WCP-RL, and WCP-LSL). Because we used proportions values, an arcsine square root transformation was calculated using SPSS (equation 1, SPSS version 20, IBM). The equation is based on the raw data and includes a Bartlett substitution [62] of 0 by 1/4n as well as a correction for converting radians to degrees; conversion factor 57.295 [63].


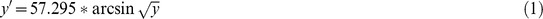


Following the arcsine transformation, a one-tailed χ^2^ test including Yates correction (SsS Rubisoft Software, Germany) was used for statistical analysis.

Permission number of our observational study on-farm was approved by Rhein-Kreis Neuss (Az.: 39.1-21-50), according to § 4 Abs. 3 TierSchG for scientific purposes. This study does not contain any animal experiments that needed ethical approval since the normal behavior of domesticated animals was observed within their natural environment. There was no interference caused by humans. Any requirements by the animals such as food, water, housing and free range were available unlimited. Observations were made with the permit according to § 11 Abs. 1 Nr. 1 b of the German Protection of Animals Act.
